# Position paper of the Italian Association of Medical Diabetologists (AMD), Italian Society of Diabetology (SID), and the Italian Study Group of Diabetes in pregnancy: Metformin use in pregnancy

**DOI:** 10.1007/s00592-023-02137-5

**Published:** 2023-07-04

**Authors:** Laura Sciacca, Cristina Bianchi, Silvia Burlina, Gloria Formoso, Elisa Manicardi, Maria Angela Sculli, Veronica Resi

**Affiliations:** 1 Interassociative Diabetes and Pregnancy Study Group, Italian Association of Medical Diabetologists (AMD), Italian Society of Diabetology (SID), Rome, Italy; 2 Department of Clinical and Experimental Medicine, Endocrinology Section, University of Catania, Catania, Italy; 3 Metabolic Diseases and Diabetes Unit, University Hospital of Pisa, Pisa, Italy; 4 Department of Medicine, DIMED, University of Padova, Padova, Italy; 5 Department of Medicine and Aging Sciences, Center for Advanced Studies and Technology (CAST, Ex CeSIMet) G. d’Annunzio University Chieti-Pescara, Chieti, Italy; 6 Diabetes Unit, Primary Health Care, Local Health Authority of Reggio Emilia-IRCCS, Reggio Emilia, Italy; 7 Endocrinology and Diabetes, Bianchi-Melacrino-Morelli Hospital, Reggio Calabria, Italy; 8 Endocrinology Unit, Fondazione IRCCS Ca’ Granda Ospedale Maggiore Policlinico, Milan, Italy

**Keywords:** Gestational diabetes, Type 2 diabetes, Obesity, Polycystic ovary syndrome, Placenta, Neonatal outcomes

## Abstract

**Objective:**

This document purpose is to create an evidence-based position statement on the role of metformin therapy in pregnancy complicated by obesity, gestational diabetes (GDM), type 2 diabetes mellitus (T2DM), polycystic ovary syndrome (PCOS) and in women undergoing assisted reproductive technology (ART).

**Methods:**

A comprehensive review of international diabetes guidelines and a search of medical literature was performed to identify studies presenting data on the use of metformin in pregnancy. The document was approved by the councils of the two scientific societies.

**Results:**

In condition affecting the fertility, as PCOS, metformin use in pre-conception or early in pregnancy may be beneficial for clinical pregnancy, even in ART treatment, and in obese-PCOS women may reduce preterm delivery. In obese women, even in the presence of GDM or T2DM, metformin use in pregnancy is associated with a lower gestational weight gain. In pregnancy complicated by diabetes (GDM or T2DM), metformin improves maternal glycemic control and may reduce insulin dose. Neonatal and infant outcomes related to metformin exposure in utero are lacking. Metformin use in women with GDM or T2DM is associated with lower birth weight. However, an increased tendency to overweight–obesity has been observed in children, later in life.

**Conclusions:**

Metformin may represent a therapeutic option in selected women with obesity, PCOS, GDM, T2DM, and in women undergoing ART. However, more research is required specifically on the long-term effects of in utero exposition to metformin.

## Introduction

Treatment of hyperglycemia in pregnancy is mandatory to reduce the risk of adverse maternal and fetal/neonatal outcomes. Metformin use in pregnancy is still a controversial topic. It is well known that metformin improves maternal glycemic control, and it is associated with reduced gestational weight gain in women with T2DM [1]. However, there are relatively a few data about the long-term effects of metformin on offspring of mother with hyperglycemia in pregnancy. It has been reported that neonates born to metformin-treated mothers had lower weight compared to those whose mothers were treated with insulin during pregnancy; however, childhood body mass index (BMI) was significantly higher in the metformin-exposed group [2].

Recently, the Italian Medicines Agency (AIFA) has modified the therapeutic indication (summary of product characteristics [SmPC]) for metformin hydrochloride tablets both immediated release and extended release relatively the use in pregnancy and in the periconceptional period. The SmPC indicates that "if clinically appropriate, the use of metformin may be considered during pregnancy and in the periconceptional period in addition or as an alternative to insulin therapy"[3–6]. The purpose of this document is to review the evidence-based literature regarding the use and the short- and long-term effects of metformin in pregnancies complicated by obesity, GDM, T2DM, PCOS and in women undergoing ART to create an evidence-based position statement on the role of metformin therapy in pregnancy. In particular, the impact of the use of metformin during pregnancy on maternal, fetal, and neonatal outcomes as well as the long-term effects on offspring was evaluated.

An Italian Editorial Board composed of experts involved in the clinical management of hyperglycemia in pregnancy was enlisted to review the published data, draw up supporting assertions and conclusions, and make clinical recommendations on the use of metformin in pregnancy.

For this position statement, a comprehensive review of international consensus was conducted, and a search of the medical literature was performed to identify studies presenting data on the use of metformin in pregnancy. Priority was given to randomized controlled trials (RCTs) as well as to systematic reviews and meta-analyses of such trials and Cochrane reviews. The references list provided is not exhaustive as a systematic literature review; the position statement provides pertinent publications relevant to the topic. Further updates will be considered periodically according to the developments in evidence-based literature.

## Mechanism of action of metformin

Metformin is a synthetic analogue of guanidine belonging to the biguanide family. It is the most prescribed oral hypoglycemic agent for type 2 diabetes worldwide, due its efficacy on glycemic control alone or in combination with many other antidiabetic drugs [7]. Metformin belongs to the class of insulin sensitizers and exerts its glucose-lowering effect primarily by decreasing hepatic glucose production through suppression of gluconeogenesis, and a modest effect to increase insulin-stimulated peripheral glucose uptake in the other target tissues of insulin action [8, 9]. The hepatic effect of metformin causes a decrease in hepatic glucose production which results in a reduction of blood glucose levels, in particular the fasting plasma glucose (FPG), associated with a marked decline of hyperinsulinemia. Furthermore, metformin increases intestinal glucose uptake and stimulates the secretion of glucagon-like peptide-1 (GLP-1) [8, 9]. The increase in GLP-1 in turn may increase the secretion of insulin and decrease the glucagon one and could also contribute to weight loss. Finally, metformin induces changes in the gut microbiome, which could influence glucose metabolism [8, 10]. Although the exact mechanism of action of metformin is incompletely understood, it exerts its effect primarily on the mitochondria to reduce hepatic gluconeogenesis and glucagon-mediated function. These effects are obtained by the reduction of mitochondrial ATP production, the consequent increase of hepatic levels of AMP, and the activation of AMP-activated protein kinase (AMPK), an important sensor of nutrient availability, which stimulates the catabolic pathways (greater oxidation of fatty acids) and inhibits the anabolic pathways (gluconeogenesis, protein synthesis, fatty acid synthesis). Metformin enters in the mitochondrion through the organic cation transporter 1 (OCT1) channel expressed on the hepatocyte membrane [9].

## Transport of metformin across the human placenta

Metformin, unlike insulin, can cross the human placenta. In metformin-exposed pregnancies, by serum samples from umbilical cord, placental, and fetal tissues, they have demonstrated that metformin has similar concentrations in the maternal and fetal circulation, suggesting active transport of metformin from the maternal circulation across the placenta and into fetal tissues [11, 12]. One of the mechanisms involved in mother-fetal placental passage appears via the organic cation transporters (OCT) [13, 14]. Human placentas express several OCT isoforms (OCT1, OCT2, OCT3, MATE1 and MATE2) [15]. Several studies have reported the expression on the maternal interface of the placenta at the syncytiotrophoblast apical membrane of norepinephrine transporter (NET), serotonin transporter (SERT), and organic cation transporter novel type 2 (OCTN2). OCT3, localized on the fetal interface of the placenta at the syncytiotrophoblast basal membrane, has been demonstrated to be the key transporter for fetal metformin [14]. Placental OCT3 expression increases with gestational age. Thus, it is hypothesized that high concentrations of metformin can reach fetal tissues only in the late gestation [14]. Moreover, whether fetal tissues express OCTs have not been carefully studied, data on the immediate effects of metformin on placental function and fetus development are scarce. Concerns regard the increased embryonic AMPK activity that may play roles in diabetic embryopathy. Stimulation of AMPK inhibits expression of Pax3, a gene that is essential for neural tube closure, and this may induce neural tube defects (NTD). However, animal studies do not suggest an increase in embryopathy with early metformin exposure in vivo, although the same authors concluded that further studies are necessary to determine the effects on overall embryo development [15]. Compared to the embryo, both fetal and placental cells are more dependent on oxidative metabolism and mitochondrial activity. Metformin increases the AMP:ATP ratio that stimulates AMPK activity. AMPK regulates several processes including gene expression, protein synthesis and cell growth. The most thoroughly described mechanism by which AMPK regulates cell growth is via suppression of the mammalian target of rapamycin complex 1 (mTORC1) pathway. Metformin may affect fetal development for the inhibition of mTOR pathway, cell proliferation and mitochondrial function [16]. However, the exposure to metformin during the first trimester of pregnancy, evaluated in a case–control study, does not seem to increase the risk of all non-genetic congenital anomalies [17]. Metformin is associated with maternal vitamin B12 deficiency and antifolate like activity. Folate: vitamin B12 imbalance induced by metformin may lead to genomic instability and aberrant gene expression, which could potentially influence fetal programming [14]. Women treated with metformin in pregnancy routine screening for vitamin B12 deficiency may therefore need to be considered, and in long-term metformin users as in T2DM, or in all conditions of deficiency, a supplementation with a multivitamin supplement may be indicated for a protective effect for the fetus. Vitamin B12 and folic acid supplementation may be an effective prophylactic approach to reduce the adverse effects of metformin on offspring. Although further studies are needed to elucidate the appropriated dosage.

## Metformin in pregnancy complicated by obesity and overweight: maternal outcomes

The prevalence of obesity, defined as a body mass index (BMI) ≥ 30 kg/m^2^, has increased significantly in recent decades, becoming more common during pregnancy as known women with obesity are more likely than women of normal weight to experience complications like gestational diabetes and/or high blood pressure during pregnancy. They are also at increased risk of a cesarean section or maternal complications [18, 19].

Improving diet and increasing exercise have had a very small effect on complications related to pregnancy. Metformin has been evaluated in a number of trials of obese pregnant women to assess the pregnancy outcome. In the first randomized, double-blind, placebo-controlled trial, the EMPOWaR, 449 women were randomized stratified by study site and BMI quartiles (30–39 vs. ≥ 40 kg/m^2^) to either placebo or metformin (500 mg increasing to a maximum of 2500 mg) daily from between 12 and 16 weeks of gestation until delivery of the baby [20]. The average gestational weight gain (GWG) in women treated with metformin was 6.7 kg which is within the Institute of Medicine (IOM) recommendations of 5–9.1 kg for women with BMI ≥ 30 kg/m^2^ and was not statistically different to the GWG seen in women receiving placebo [21]. About maternal outcome, 18% of women treated with metformin developed GDM compared to 24% in the placebo group (non-significant) and no reduction in preeclampsia rate. The authors’ conclusion was a negative indication for the use of metformin in obese women without diabetes to improve the pregnancy outcome. In the study of El Fattah, a prospective study with two hundred participants with a BMI of ≥ 35 kg/m^2^, pregnant women in the early second trimester were randomized to metformin 500 mg twice a day and placebo [22]. There was a significant reduction in the GWG in the metformin group compared to the other group where the mean was 6.5 kg for the metformin group and 11.6 kg for the placebo one. There was not a significant difference in the occurrence of pregnancy complications as gestational diabetes and hypertension. A recent double-blind, placebo-controlled trial (the Metformin versus Placebo in Obese Pregnant Women without Diabetes Mellitus, MOP study) included pregnant women with a BMI of more than 35 kg/m^2^ to receive metformin, at a dose of 3.0 g per day, or placebo (*n* = 225 women in each group) from 12 to 18 weeks of gestation until delivery [23]. The metformin use was associated with a reduction in GWG (4.3 kg compared to 6.1 kg in the placebo group; *p* < 0.001) as was the incidence of preeclampsia (3.0% vs. 11.3%; odds ratio 0.24; 95% confidence interval 0.10 to 0.61; *p* = 0.001). However, by the authors’ own admission, the study was underpowered to evaluate the last outcome. A 2018 Cochrane review included these three studies and the outcomes were available for 1034 participants: Women who received metformin may have a slightly lower gestational weight gain (GWG) [mean difference (MD) − 2.60 kg, 95% CI − 5.29 to 0.10; *n* = 899 women] and probably makes little or no difference in the risk of women developing gestational diabetes as reported [Risk Ratio (RR) 0.85, 95% CI 0.61–1.19; 3 studies, 892 women] [24]. Metformin may have little difference in the risk of women developing gestational hypertension (average RR 1.02, 95% CI 0.54–1.94; 3 studies, 1040 women) or preeclampsia (RR 0.74, 95% CI 0.09–6.28; 2 studies, 840 women) [24]. The more recent GRoW randomized, doubled-blind study examined the use of metformin (to a maximum dose of 2000 mg/day) compared to placebo in 500 women with a BMI of ≥ 25 kg/m^2^ at 10–20 weeks’ gestation [25]. Women receiving metformin had lower average weekly gestational weight gain (adjusted mean difference − 0.08 kg, 95% CI 0.14–0.02; *p* = 0.007) and were more likely to have GWG below recommendations [adjusted risk ratio (aRR) 1.46, 95% CI 1.10–1.94; *p* = 0.008), but total GWG did not differ significantly between groups. The rates of GDM were unchanged between the two groups. The author’s interpretation was that metformin does not improve maternal outcomes. Adverse effects included abdominal pain, diarrhea, or headache were reported in women receiving metformin compared with women receiving placebo cumulatively (RR 1.63, 95% CI 1.27–2.08; 1 study, 400 women) [24]. In a recent review by Dunne about the metformin in pregnancy, all the different results in maternal outcomes observed in these RCTs were attributable to patient characteristics: all Caucasian women in the EMPOWaR study whereas the MOP trial had more ethnic diversity; in the GRoW study the BMI was 32.5 kg/m^2^, compared to 38 kg/m^2^ in EMPOWaR and 38.6 kg/m^2^ in MOP and in the medication dosage (the maximum dose of metformin was 2500 mg in the EMPOWaR, in the MOP trial 3000 mg, and in the GRoW study 2000 mg) [26]. We did not identify studies of metformin in combination with another treatment in obese women in pregnancy. Women with obesity in pregnancy may represent a heterogeneous group and at now the evidence to suggest that metformin improves maternal outcomes in obesity without diabetes, as the rates of GDM and preeclampsia, are too small. Very obese women treated with metformin in pregnancy may have a slightly lower GWG.

In Table 1 are summarized the effects on maternal outcomes of metformin compared to control group in overweight or obese women.

**Table 1:** Maternal outcomes in overweight or obese women treated with metformin during pregnancy

	Overweight women	Obese women
Total GWG	No significant difference	Reduced
GDM rate	No significant difference	No significant difference
Gestational hypertension/Preeclampsia rate	No sufficient data	Reduced
Caesarian section	Reduced	No significant difference
Preterm delivery (< 37 weeks)	No sufficient data	No significant difference

GWG, Gestational weight gain; GDM, Gestational diabetes

## Metformin in pregnancy complicated by obesity: fetal and long-term offspring outcomes

The infants of obese women are more likely to experience requiring admission to the neonatal unit or intensive care, or distress respiratory syndrome immediately after birth [18]. Unfortunately, there is a paucity of long-term follow-up data on the infants of obese women exposed to metformin.

### Short-term neonatal outcomes

In the EMPOWaR study, the mean birth weight at delivery was 3463 g (± 660) in the placebo group and 3462 g (± 548) in the metformin group (the proportion of liveborn babies weighing more than the 90th percentile was 38 [17%] of 220 and 31 [14%] of 214 babies, respectively) with no statistical difference [20]. In the MOP study, the primary outcome of the study was the reduction in neonatal birth weight but there was no significant between-group difference in the median neonatal birth weight z score (0.05 in the metformin group and 0.17 in the placebo group; *p* = 0.66) [23]. In the GRoW study, there was no significant difference in the proportion of infants with birth weight greater than 4000 g (40 [16%] with metformin vs. 37 [14%] with placebo); (aRR 0.97, 95% CI 0.65–1.47; *p* = 0.899) or born Large for gestational age (LGA) or small for gestational age (SGA) [25].

### Long-term infant outcomes

The EMPOWaR follow-up has been reported infant outcomes in body composition, peripheral blood pressure, arterial pulse wave velocity and central hemodynamics in infants at a mean age of 5 years old, and no differences were found in any parameters in the children born to mothers exposed to metformin (*n* = 19) versus placebo (*n* = 21) in pregnancy [27]. The MOP trial followed up 151 children (77 exposed to metformin prenatally) at 3.9 ± 1.0 years, and there was no significant difference in metabolic profile and body composition. Compared to the placebo group, infants exposed to metformin had significantly lower central hemodynamic function (mean adjusted decrease, − 0.707 mmHg for aortic systolic blood pressure, − 1.65 mmHg for aortic pulse pressure and − 2.68% for augmentation index) and lower left ventricular diastolic function (adjusted difference in left atrial area, − 0.525 cm^2^ in isovolumic relaxation time, − 0.324 ms and in pulmonary venous systolic wave, 2.97 cm/s). Children of obese mothers who were prenatally exposed to metformin have improved central hemodynamic and cardiac diastolic indices [28]. The follow-up of children born to women who participated in the GRoW trial at six months, 18 months, and three to five years of age was recently published. Child BMI *z*-score > 85th centile was similar for children born to women who received metformin compared with women who received placebo at all of the time points measured, and the effect of treatment did not differ over time. In both groups, the proportion of children with BMI *z*-score > 85th centile increased over time from approximately one-third of infants at six months of age, to at least half of children at 18 months, and three to five years of age. Despite the lack of differences observed between treatment groups, this cohort of children remains at high risk of overweight and obesity, with approximately half of children assessed having BMI above the 85th centile for age and sex, a figure almost double that observed in the general Australian childhood population [29]. Women who received metformin or placebo had a similar risk of their baby being LGA cumulatively (RR 0.95, 95% CI 0.70–1.30; 2 studies, 831 infants; high-quality evidence), and there were no other important differences identified for other infant outcomes: stillbirth and neonatal death; hypoglycemia; hyperbilirubinemia or shoulder dystocia [24]. We do not know whether preconception use of metformin at different dosage would have been effective in some neonatal outcomes in obese women. Women who receive metformin due to obesity or overweight do not seem to demonstrate any change in infant outcomes. Initial mid-term follow-up data on metformin-exposed children seem comforting but long-term follow-up studies are needed to evaluate any benefit or unrecognized impact of intrauterine metformin exposure.

In Table 2 are summarized the effects on neonatal and infant outcomes of metformin compared to control group of overweight or obese women. Table 3 shows main maternal and infant outcomes in overweight or obese women treated with metformin during pregnancy.

**Table 2:** Neonatal and infant outcomes in overweight or obese women treated with metformin during pregnancy

	Overweight women	Obese women
**Newborns**		
Birth weight	No significant difference	No significant difference
LGA/Macrosomia	No significant difference	No significant difference
SGA	No significant difference	No significant difference
Hypoglycemia	No significant difference	No significant difference
Intensive care unit admission	No significant difference	Reduced
**Infants**		
BMI	Higher	No significant difference
Total body fat	No significant difference	No significant difference
Systolic blood pressure	No significant difference	Reduced

LGA, large for gestational age; SGA, small for gestational age; BMI, body mass index

**Table 3:** Main maternal and infant outcomes in overweight/obese women treated with metformin during pregnancy

	Maternal outcomes	Infant outcomes
**Obesity**	Slightly lower gestational weight gain (GWG) in very obese women	No apparent effect in mid-term
**Overweight**	Lower average weekly gestational weight gain	No evidence of any beneficial effects of in-utero exposure to metformin among infants and children born to women with overweight in mid-term follow-up

## Metformin in PCOS women: maternal outcomes

Polycystic ovary syndrome is the most common hormonal disorder in women and accounts for about 80% of women with anovulatory infertility. Pregnancy in women with PCOS is associated with a higher incidence of GDM (40% to 50%) and related fetal macrosomia, gestational hypertensive disorders (such as preeclampsia and pregnancy-induced hypertension) (5%), as well as induction of labor, cesarean section, preterm birth [30, 31]. The association of PCOS with pregnancy and birth complications varies by PCOS phenotype, ethnicity, history of metabolic disorders and women's lifestyle. New evidence provided in the last 10 years suggests potential benefit of metformin treatment in pregnancies of women affected by this syndrome. These benefits could be mediated by the positive metabolic, endocrine, vascular and anti-inflammatory properties which modulate the main factors involved in first trimester abortion in these patients [8, 32–36].

A small prospective, randomized, double-blind, placebo-controlled pilot study was published in 2004 by Venky & coll. Forty pregnant Norwegians women with PCOS were randomized to either metformin 850 mg twice daily or placebo. Primary outcome was the change in serum androgens levels while secondary outcome measures were pregnancy complications and outcomes. Metformin treatment did not reduce maternal androgen levels. However, in the metformin group no severe pregnancy complication occurred, while 22% of pregnant in the placebo group (*p* = 0.01) experienced severe adverse outcomes (pre-term delivery and 1 case of pelvic deep vein thrombosis and massive lung embolism) [37]. Based on these observations, the same authors conducted the first RCT in women with PCOS published in 2010. Metformin 2000 mg was started during the first trimester of pregnancy in 247 Caucasian women diagnosed with PCOS using the Rotterdam criteria; about 30% of women received metformin before the enrollment in the trial. Primary endpoints were the prevalence of preeclampsia, preterm delivery, GDM, and a composite of these three diagnoses. Any difference in the prevalence of preterm delivery (3.7% metformin group versus 8.2% placebo; − 4.4%; 95% CI − 10.1 to 1.2; *p* = 0.12), preeclampsia (7.4% metformin vs. 3.7% placebo; 3.7%; 95% CI − 1.7to 9.2; *p* = 0.18) or GDM (17.6% metformin vs. 16.9% placebo; 0.8%; 95% CI − 8.6 to 10.2; *p* = 0.87) was observed between treatment groups. The composite primary endpoint prevalence was 25.9% and 24.4%, respectively (1.5%; 95% CI − 8.9 to 11.3, *p* = 0.78) [38]. It must be noted that metformin treatment was associated with a lower GWG (− 2.2 kg). Moreover, a per-protocol analysis revealed a reduction in preterm delivery in metformin treated women (from 10 to 2.8%; 95% CI, 0.9–13.9; *p* < 0.05). The study did not assess pregnancy loss and it lacked the statistical power to detect changes in the occurrence of GDM [38].

The PregMet2 was a randomized, placebo-controlled, double-blind, multicenter trial conducted in Norway, Sweden, and Iceland including 487 (244 in the metformin and 243 in the placebo group) singleton pregnant women with PCOS [39]. Women received metformin 2000 mg/day from the late first trimester until delivery and the primary outcome was the composite incidence of late miscarriage (between week 13 and week 22 and 6 days) and preterm birth (between week 23 and week 36 and 6 days). Secondary endpoints included the incidence of gestational diabetes, preeclampsia, pregnancy-induced hypertension, and admission of the neonate to the neonatal intensive care unit. In the intention-to-treat analysis, the composite primary outcome of late miscarriage and preterm birth occurred in 12 (5%) of 238 women in the metformin group and 23 (10%) of 240 women in the placebo group [odds ratio (OR) 0.50, 95% CI 0.22–1.08; *p* = 0.08]. No significant differences for our secondary endpoints, including incidence of gestational diabetes [60 (25%) of 238 women in the metformin group vs. 57 (24%) of 240 women in the placebo group; OR 1.09, 95% CI 0.69–1.66; *p* = 0.75], were found. In order to increase the study power, the authors performed a post hoc individual participant data analysis of pregnancy outcomes, merging data from the PregMet2 trial with the two previous randomized control trials published by Venky in 2004 and 2010. The analysis included data from nearly 800 women and showed a significant improvement in the primary outcome which was the composite incidence of late miscarriage and preterm birth (OR 0.43, 95% CI 0.23–0.79; *p* = 0.004) [37, 40]. The improvement was more evident in obese women (BMI ≥ 30 kg/m^2^) and in those who underwent assisted reproduction technology (ART). It must be considered that the main driver of these positive results was the reduction in preterm delivery (from 8 to 4%; *p* < 0.05) [25]. Importantly, these observations were homogeneous in term of phenotype, severity, and mode of conception in the participants presenting a broad spectrum of PCOS. However, metformin had no effect on the incidence of GDM (OR 1.06, 95% CI 0.68–1.64; *p* = 0.83) or on the need for insulin treatment (1.00, 0.35–2.93; *p* = 1.00) [39].

In 2021 a meta-analysis including six randomized control trial studies involving 1229 participants assessing metformin's effects on pregnancy outcomes in women with polycystic ovary was published.

Metformin administration in PCOS pregnancies was associated with reduced preterm delivery risk (RR 0.45; 95% CI 0.25–0.80; *p* = 0.007) and with larger neonatal head circumference (MD 0.47; 95% CI 0.20–0.74; *p* = 0.0006) but had no effect on the incidence of GDM, miscarriage and preeclampsia [41].

A recent published nationwide population-based cohort study in Swedish population including 1,016,805 singleton births from 686,847 women support the possible benefit of metformin revealing that women with PCOS not using metformin during pregnancy presented higher risks of preeclampsia (OR 1.09, 1.02–1.17), gestational diabetes (OR 1.71, 1.53–1.91), cesarean section (OR 1.08, 1.04–1.12) and preterm birth (OR 1.30, 1.23–1.38) [42].

Based on the available studies, a recent published expert opinion on the clinical management of pregnancy in women with polycystic ovary syndrome suggested to consider metformin only as a pre‐pregnancy treatment for cardiometabolic risk factors and weight management [43]. Nevertheless, from the trials so far discussed emerged a possible benefit of the early treatment with metformin started from the first trimester and continued throughout the pregnancy. No safety concerns in metformin treated patients arise, with no evidence of teratogenicity.

In Table 4 are summarized the effects on maternal outcomes of metformin compared to control group in PCOS women.

**Table 4:** Maternal outcomes in PCOS women treated with metformin during pregnancy

	PCOS Women
Fertility	Increased
Total GWG	Reduced
GDM rate	No significant difference
Gestational hypertension/Preeclampsia rate	No significant difference
Caesarian section	No significant difference
Miscarriage/Preterm delivery (< 37 weeks)	Reduced

GWG, Gestational weight gain; GDM, Gestational diabetes

## Metformin in PCOS women: fetal and long-term offspring outcomes

While common features of PCOS (overweight or obesity) are recognized as risk factors for fetal and birth complications, PCOS per se is not consistently perceived as a risk factor for fetal/neonatal health in pregnant women with this syndrome [43]. Possible adverse outcomes to be monitored in PCOS mothers could be low or high birth weight, low Apgar scores and higher rate admission to Neonatal Intensive Care Unit (NICU) [43, 44].

### Short-term neonatal outcomes

Exploring available data regarding possible complications in fetus exposed to metformin during pregnancy the important information derives from the trial discussed above, from which result that metformin treatment in PCOS pregnant seems not to affect the incidence in birth weight, birth length, ponderal index, APGAR score, umbilical artery pH, placenta weight, intrauterine/neonatal death, and intensive care unit admission [37–39]. The only differences emerged in term of head circumference which was significantly larger in the metformin group than in the placebo group (35.6 cm in metformin vs. 35.2 cm in placebo group—mean difference 0.4, 0.2 to 0.6; *p* = 0.0004). However, this effect could be modified by maternal pre-pregnancy BMI as evidenced by a sub-study of the PregMet trial [37–39]. A meta-analysis by Cao & coll. based on six randomized control trial studies involving 1229 participants confirmed that metformin administration in PCOS pregnancies was associated with larger neonatal head circumference (MD 0.47; 95% CI 0.20–0.74; *p* = 0.0006) without significant effect of metformin on the incidence of neonatal length and birth weight [41]. The benefit of metformin treatment is shown by a large nationwide population-based cohort study conducted in Swedish which underlines how babies of women with PCOS untreated with metformin during pregnancy presented low birth weight (OR 1.29, 1.20–1.38), low Apgar scores (OR 1.17, 1.05–1.31) and were LGA (OR 1.11, 1.03–1.20) as compared to babies from metformin treated mothers [42].

### Long-term infant outcomes

Data regarding the offspring of women enrolled in the pilot study by Venky & coll. as well as in the PregMet trial were collected and analyzed by the same authors. At age of 4 years, there were more overweight/obese children in the metformin group [26 (32%) than in the placebo group 14 (18%); OR 2.17, 1.04 to 4.61; *p* = 0.038] [40]. This observation was confirmed by the follow-up of children from the PregMet study at the age of 10 years. Offspring of women from the metformin group had a higher BMI Z score than those of placebo group (difference in means = 0.41, 95% CI 0.03–0.78; *p* = 0.03) [45]. However, it must be pointed out that the risk of obesity in the offspring correlated with maternal pre-pregnancy BMI. Moreover, no increase in cardiometabolic risk factors (such as C-peptide and cholesterol) was observed. A recent meta‐analysis supports the fact that children born from women with GDM or PCOS using metformin during pregnancy presented higher long‐term weight [46].

The effect of metformin use in PCOS women during pregnancy on childhood obesity requires further exploration, also considering data from the recent Swedish nationwide cohort study. From the analysis emerged a positive association between PCOS and childhood obesity (HR 1.61, 95% CI 1.44–1.81). On the other hands no association resulted between maternal metformin consumption and obesity in the offspring of PCOS women while the conjunction emerged in non-PCOS metformin treated mothers (HR 1.67, 95% CI 1.05–2.65) [42].

The described evidence depones for a possible benefit of metformin on fetal and neonatal complications. However, infants of women treated with metformin during pregnancy could be heavier and more likely to be overweight or obese which indicate a potential risk of unfavorable cardiometabolic, therefore this issue needs further analysis.

In Table 5 are summarized the effects of metformin on neonatal and infant outcomes compared to control group of PCOS women. Table 6 shows main maternal and infant outcomes in PCOS women treated with metformin during pregnancy.

**Table 5:** Neonatal and infant outcomes in PCOS women treated with metformin during pregnancy

	PCOS women
**Newborns**	
Birth weight	No significant difference
Macrosomia	No significant difference
Hypoglycemia	No significant difference
Neonatal intensive care unit admission	No significant difference
**Infants**	
BMI	Higher
Total body fat	No significant difference
Systolic blood pressure	No significant difference

BMI, body mass index

**Table 6:** Main maternal and infant outcomes in metformin treated women with PCOS

	Maternal outcomes	Infant outcomes
**Polycystic ovary syndrome**	Reduction in preterm delivery No significant effect on rates of GDM	Overweight or obesity (at age 4 to 10 years) are more common in infant of women with a history of PCOS treated with Metformin during pregnancy No apparent increased cardiovascular risk

## Metformin in pregnancy obtained by assisted reproduction technology

Assisted reproduction could be an active treatment option for infertility in women with PCOS who do not respond to ovulation induction treatments. After half a century, metformin has established itself as a first defense against insulin-dependent morbidities and undoubtedly has become a useful drug for improving fertility outcomes in both male and female patients. The ASRM/ESHERE consensus recommended restricting the use of metformin to women with glucose intolerance [47]. However, it is recorded that metformin has commonly been administered as a co-treatment with controlled ovarian stimulation (COH) in women with PCOS who undergo in vitro fertilization (IVF) [48]. In 2006 Tang and coll. explored the effect of metformin in women with PCOS undergoing IVF by a large randomized, placebo-controlled, double-blind trial. The primary outcome was an improvement in the overall fertilization rate [49]. A short course of 28 days of metformin during the IVF cycle had not any effects on the total dose of rFSH required per cycle, on the median number of oocytes retrieved per cycle (metformin = 17.2, placebo = 16.2; *p* = 0.459) and either on the overall fertilization rates (metformin = 52.9%, placebo = 54.9%; *p* = 0.641). However, both the clinical pregnancy rates beyond 12 gestation weeks per cycle (metformin = 38.5%, placebo = 16.3%; *p* = 0.023) and per embryo transfer (metformin = 44.4%, placebo = 19.1%; *p* = 0.022) were significantly improved in women treated with metformin with a significant decrease in the incidence of severe ovarian hyperstimulation syndrome (OHSS) (metformin = 3.8%, placebo = 20.4%; *p* = 0.023), which was still significant after adjustment for BMI, total rFSH dose and age (OR = 0.15; 95% CI 0.03, 0.76; *p* = 0.022) [49]. Metformin has also been evaluated in combination with clomiphene citrate (CC). In a large Dutch multicenter trial, 228 women with PCOS were randomly allocated to receive either CC plus metformin or CC plus placebo [50, 51]. There was no difference in the ovulation rate in the metformin group compared with placebo (64% vs. 72%), neither in the rates of ongoing pregnancy (40% vs. 46%) nor miscarriage (12% vs. 11%) [51]. Similar results were reached by Legro and coll. in 2007 [52].

A recent Cochrane review included 13 randomized controlled trials involving a total of 1132 women assigned to receive either metformin (*n* = 570) or placebo/no treatment (*n* = 563) aimed to explore the effect of metformin treatment before and during IVF or Intracytoplasmic Sperm Injection (ICSI) in women with PCOS. In most of the studies metformin was used before and during ovarian stimulation for IVF or ICSI treatment at the dosage of 1000 mg/day. The analysis evidenced that as compared to placebo/no treatment, metformin seemed to have no clear benefit in term of live birth rates (RR 1.30, 95% CI 0.94–1.79; *p* = 0.12). However, it may increase clinical pregnancy rates in association to long protocol GnRH agonist (RR 0.48, 95% CI 0.29–0.79; *p* = 0.004) and may reduce the incidence of OHSS (RR 0.40, 95% CI 0.26–0.60; *p* < 0.0001). The results on miscarriage rate are uncertain [53]. Promising results come from a recent systematic review and meta-analysis of Unanyana and coll. comparing metformin, inositol or metformin plus inositol treatment with placebo or no treatment in women with PCOS who underwent assisted reproductive technologies (ovulation induction/IVF/ICSI/IUI). Primary outcomes were clinical pregnancy rate (CPR), and the incidence of OHSS; the secondary outcomes was live birth rate (LBR). A total of 35 reports were included. According to the results obtained CPR were significantly higher in the metformin group as compared to placebo (RR 1.30, 95% CI 1.12–1.50; *p* = 0.0004) while the OHSS occurred more frequently in the controls as compared to metformin treated patients (RR 0.34, 95% CI 0.17–0.69; *p* = 0.003); no statistically significant difference was found regarding LBR (RR 1.12, 95% CI 0.93–1.36; *p* = 0.24) [54].

Metformin could be considered a therapeutic option to improve fertility and to reduce OHSS incidence in women with PCOS undergoing ART (Table 7).

**Table 7:** Main maternal and infant outcomes in women with PCOS treated with metformin during assisted reproduction technology procedure

	Maternal outcomes	Infant outcomes
Pregnancy obtained by assisted reproduction technology	Fertility improvement in women with PCOS resistant to clomiphene citrate Lower rate of ovarian hyperstimulation syndrome (OHSS) in women with PCOS	No available data

## Metformin in pregnancy complicated by GDM: maternal outcomes

GDM is defined as diabetes diagnosed in the second or third trimester of pregnancy that was not clearly overt diabetes prior to gestation. If it is not properly diagnosed and treated, GDM causes short- and long-term cardiometabolic complications both for mother and child.

Insulin is the preferred medication for GDM treatment when diet and physical activity are not sufficient to obtain a good metabolic control. However, international guidelines defined metformin as a valid option, but its use remains area of controversy and doubt in the treatment of GDM, especially regarding the long-term effect in the childhood. In this contest, the metformin in gestational diabetes (MiG) trial prospectively compared pregnancy outcomes in more than 700 women with GDM randomized to either metformin (*n* = 363) or insulin treatment (*n* = 370) [55]. Mean BMI at enrollment was 35 kg/m^2^. Metformin was started at a dose of 500 mg once or twice daily and increased to meet glycemic targets up to a maximum daily dose of 2500 mg. If the targets were not achieved with metformin alone, insulin was added. The primary outcome was a composite of neonatal outcomes. Secondary outcomes included neonatal anthropometric measurements, maternal glycemic control, maternal hypertensive complications, postpartum glucose tolerance, and acceptability of treatment.

The overall mean maternal 2-h postprandial glucose levels were slightly lower in the metformin group, with not differences in the 2 weeks before delivery. In the metformin-treated obese women, there was a significant reduction in GWG. It is noteworthy that 46% of women treated with metformin also required insulin to achieve a good metabolic control even if their insulin requirements were significantly lower than those treated with insulin alone. In addition, women in the metformin group had less weight gain between the time of enrollment and 36 weeks of gestation than did women in the insulin group. Maternal hypertensive complications and postpartum glucose tolerance did not differ between the two groups [55].

Taking in consideration all this data on maternal outcomes, we can consider that patients obese and very obese with GDM could benefit from metformin treatment in order to reduce the gestational weight gain. In particularly, severe obese GDM women insulin treated, metformin use may reduce the insulin dose.

In Table 8 are summarized the effects on maternal outcomes of metformin compared to control group in GDM women.

**Table 8:** Maternal outcomes in GDM women treated with metformin during pregnancy

	GDM women
Glycemic control	Comparable to insulin
Total insulin dose	Reduced as compared to insulin alone
Total GWG	Reduced
Gestational hypertension/Preeclampsia rate	No significant difference
Caesarian section	No significant difference
Preterm delivery (< 37 weeks)	No significant difference

GWG, Gestational weight gain

## Metformin in pregnancy complicated by GDM: fetal and long-term offspring outcomes

### Short-term neonatal outcomes

In the MiG trial, the primary composite outcome evaluated a combination of neonatal hypoglycemia, neonatal respiratory distress, need for phototherapy, birth trauma, Apgar score at 5 min and birth before 37 gestational weeks. The results of the study showed no difference in the composite endpoint, but there was a significant reduction in severe neonatal hypoglycemia in the metformin group. No significant differences were found between the two groups in terms of neonatal anthropometric measures [55]. After the MiG trial, other trial evaluated the impact of treatment with metformin in pregnancy specially on infant birth weight and rates of SGA and LGA. A meta-analysis of 19 RCTs (3723 neonates) in 2019 found out that neonates born to metformin-treated mothers had significant lower birth weights (mean difference − 107.7 g, 95% CI 182.3–32.7, *I*2 = 83%; *p* = 0.005) and lower ponderal indices (mean difference − 0.13 kg/m^3^, 95% CI 0.26–0.00, *I*2 = 0%; *p* = 0.04) than neonates of insulin-treated mothers [56]. On the other side, the risk of macrosomia (OR 0.59, 95% CI 0.46–0.77; *p* < 0.001) and LGA neonates (OR 0.78, 95% CI 0.62–0.99; *p* = 0.04) were lower following maternal treatment with metformin compared to insulin.

Recently Brand and coll. evaluated if maternal pregnancy exposure to metformin is associated with increased risk of short-term adverse outcomes in the child. They found that exposure to metformin was associated with significantly lower mean birth weight, compared with insulin, in line with a significantly increased risk of SGA [5]. The increased risk of SGA associated with metformin versus insulin suggests caution in pregnancies with at-risk fetal undernutrition because one potential pathway through which metformin may influence risk of SGA is a reduced maternal food intake.

According to the available literature data, maternal exposure to metformin in women with GDM was associated to lower neonate birth weights and this could be interpreted as a positive effect related to improved glycemic control but even as a negative effect of growth restriction. In fact, the major concern about the use of metformin in the treatment of GDM is related to long-term cardio-metabolic effect in the offspring.

### Long-term infant outcomes

A follow-up study of women with GDM of the MiG study found that in the metformin group, compared with the insulin group, children at 2 years of age had larger mid-upper arm circumferences and subscapular and biceps skinfolds even if total fat mass and percentage body fat assessed by bioimpedance and DEXA were not different. So, metformin may alter fat distribution in the children exposed to metformin in utero at 2 years of age [57]. Then, in 2018, the results of 7–9 years of follow-up study of MiG offspring was published (MiG TOFU) [2]. Children were divided into 2 subgroups: the Adelaide subgroup evaluated at 7 years of age and the Auckland subgroup evaluated at 9 years of age. All the children were assessed by anthropometry, bioimpedance analysis, dual-energy X-ray absorptiometry, magnetic resonance imaging and fasting bloods. In the Adelaide subgroup, mothers were similar at enrollment as for metabolic and clinical characteristics. Women treated with metformin had higher glycemia during treatment than women randomized to insulin and neonates exposed to metformin were larger at birth respect to neonates exposed to insulin. In their offspring, at 7 years, there were no differences in all measures evaluated. In Auckland subgroup, at enrollment, women randomized to metformin had a higher BMI but gained less weight during treatment respect to women randomized to insulin. In their offspring, at 9 years, metformin group were larger by measures of weight, arm and waist circumferences, waist:height; BMI, triceps skinfold; DXA fat mass and lean mass; MRI abdominal fat volume. The potential explanation for these long-term effects of metformin exposure is provided by animal data [58]. These data show that maternal weight gain during pregnancy, maternal caloric intake during pregnancy and the gender of the infant are the main mediators of these effects. In particular, higher BMI and higher fat mass are seen in the offspring if there were caloric restriction and low maternal weight gain during pregnancy, especially in female offspring. In addition, the possible cognitive effects of metformin exposure on the offspring have been evaluated. In particular, Landi and coll. conducted a retrospective cohort study in a New Zealand population of treated women (metformin vs. insulin) linked with their child’s developmental data at age 4 years [59]. They found similar behavioral and emotional development between treatment groups. However, these data have to be confirmed by large RCT.

In Table 9 are summarized the effects of metformin compared to control group in offspring of GDM women. Table 10 shows main maternal and infant outcomes in GDM women treated with metformin.

**Table 9:** Neonatal and infant outcomes in GDM women treated with metformin during pregnancy

	GDM women
Newborns	
Birth weight	Lower
LGA/Macrosomia	Decreased
SGA	No significant difference
Hypoglycemia	Decreased
Neonatal intensive care unit admission	No significant difference
Infants	
BMI	Increased
Waist circumference	Increased

LGA, Large for gestational age; SGA, Small for gestational age; BMI, Body mass index

**Table 10:** Main maternal and infant outcomes in metformin treated women with GDM

	Maternal outcomes	Infant outcomes
**Gestational Diabetes**	Lower gestational weight gain (GWG) in obese women Reduction in insulin dose in severe obese women	Lower neonates birth weights 7–9 years old infant are heavier and with higher waist circumferences, higher waist:height ratio, higher BMI and higher fat mass volume

## Metformin in pregnancy complicated by type 2 diabetes mellitus: maternal outcomes

The increasing prevalence of T2DM in women of childbearing age continue to focus attention on the management of hyperglycemia in pregnancy [60]. Since glucose is a teratogen, optimal control of hyperglycemia is imperative from pre-conception to throughout the whole pregnancy. Currently, there is significant controversy over the best drug intervention strategy in pregnant women with diabetes, and in particular over the use of metformin. This debate is even more heated considering the need to limit gestational weight gain in women with T2DM who are often overweight or obese. Although the preferred glucose-lowering medication in pregnancy is insulin, the most common initial glucose-lowering drug for type 2 diabetes is metformin, and many women experience unplanned pregnancy while on metformin therapy. Metformin alone is typically not capable of overcoming the insulin resistance of pregnancy in T2DM and women often require high insulin doses, which are uncomfortable, costly, and associated with increased gestational weight gain.

A few studies have explored the efficacy and safety of metformin in pregnancy complicated by T2DM as alternative or adjunct therapy to insulin. Most of these studies were open-label, conducted in countries with inadequately equipped health care and were not designed to investigate differences in adverse effects between treatments.

Early small studies have documented equal glycemic control in women taking metformin compared to those taking insulin during pregnancy [61–63].

A study of 104 pregnant Ghanaian women randomized those with T2DM or GDM to either metformin or insulin between 20- and 30-weeks’ gestation [64]. The proportions of women with T2DM in each ckmangroup were 25.6% and 42.5%, respectively. Two-hour postprandial blood glucose was the primary outcome. This was significantly lower in the metformin group and only 2/52 (4%) required supplementary insulin.

A larger study, completed in Pakistan, involved 206 women with untreated T2DM randomized to receive either metformin with insulin (as necessary), or insulin alone [65]. In this study, 85% of women in the metformin group required add-on insulin, but this group experienced less maternal weight gain and less pregnancy-induced hypertension.

A meta-analysis of 16 randomized control trials comparing metformin with insulin in pregnancy in women with GDM or T2DM, concluded that metformin lowered the risk of pregnancy-induced hypertension (RR 0.56; 95% CI 0.37–0.85) and total maternal pregnancy weight gain (mean difference − 2.07; 95% CI − 2.88 to − 1.27), while not increased the risk of cesarean section (RR 0.97 95% CI 0.80–1.19) [66].

### Randomized controlled trial

The Metformin in Women with Type 2 Diabetes in Pregnancy (MiTy) trial examined the effects of metformin on maternal and neonatal outcomes in a randomized, double-masked, placebo-controlled study in T2DM and GDM involving 502 pregnant women from 29 centers in Canada and Australia [1]. Women with T2DM before pregnancy or with GDM diagnosed by 20-weeks’ gestation were treated with insulin and randomly assigned to add-on metformin (1000 mg twice daily) or placebo. Treatment was started between 6 and 22 weeks and continued to term. Compared with women in the placebo group, metformin-treated women achieved better glycemic control (HbA1c at 34 weeks' gestation 5.9% vs. 6.1%; *p* = 0.015), required less insulin (1.1 units/kg/day vs. 1.5 units/kg/day; *p* < 0.0001), gained less weight (7.2 kg vs. 9.0 kg; *p* < 0.0001) and less frequently had excessive GWG according to the IOM guidelines. Moreover, metformin treated women had fewer cesarean births (125 [53%] of 234 in the metformin group vs. 148 [63%] of 236 in the placebo group; *p* = 0.031; RR 0.85; 95% CI 0.73–0.99), without difference in hypertensive disorders (23% vs. 23%; *p* = 0.93; RR 0.99; 95% CI 0.72–1.35), diabetes complications, or maternal length of hospital stay.

Altogether, these data suggest that pregnant women with T2DM on metformin and insulin require less insulin than women treated with insulin alone. This translates into less maternal weight gain and decreased risk of maternal hypoglycemia. However, not all professional organizations agree on the use of metformin during pregnancy. For instance, the National Institute for Health and Care Excellence (NICE) declares that women with diabetes may be advised to use metformin as an adjunct or alternative to insulin in the preconception period and during pregnancy, when the likely benefits from improved blood glucose control outweigh the potential for harm [67]. On the contrary, in the American Diabetes Association (ADA) guidelines, insulin is the preferred agent for the management of glycemic control during pregnancy [6].

In Table 11 are summarized the effects on maternal outcomes of metformin compared to control group in T2DM women.

**Table 11:** Maternal outcomes in T2DM women treated with metformin during pregnancy

	T2DM women
Glycemic control	Comparable or slightly improved as compared to insulin alone
Hypoglycemia	Lower rate as compared to insulin alone
Total insulin dose	Reduced
Total GWG	Reduced
Gestational hypertension/reeclampsia rate	No significant difference
Cesarian section	Decreased
Preterm delivery (< 37 weeks)	No significant difference

GWG, gestational weight gain

## Metformin in pregnancy complicated by type 2 diabetes mellitus: fetal and long-term offspring outcomes

The first trimester is the most sensitive time in pregnancy for drug exposure because organogenesis is taking place; therefore, ideally all drugs should be avoided. However, the risks to the fetus of untreated disease are often greater than the risks of drug treatment. This is true for T2DM, in which uncontrolled hyperglycemia in utero increases the rates of miscarriage, birth defects and the complications of macrosomia [60].

Metformin crosses the placenta and might also lower glucose and insulin resistance in the fetus [68]. However, because fetal metformin concentrations are similar than those in the mother, potential safety concerns exist. Limited data are available on the outcomes of neonates by mother with T2DM treated with metformin during pregnancy.

### Short-term neonatal outcomes

In the study by Ainuddin JA and coll., the infants by mothers treated with metformin during pregnancy had less hypoglycemia and less NICU admissions > 24 h, but an increased rate of SGA [65]. The meta-analysis by Butalia, including RCT in women with T2DM or GDM treated with metformin during pregnancy, showed that metformin, compared to insulin, lowered the risk of neonatal hypoglycemia (RR 0.63; 95% CI 0.45–0.87) and LGA babies (RR 0.80; 95% CI 0.64–0.99), while not increased the risk of preterm delivery (RR 1.18; 95% CI 0.67–2.07), SGA babies (RR 1.20; 95% CI 0.67–2.14), perinatal mortality (RR 0.82; 95% CI 0.17–3.92) in a short-term follow-up period [66].

In the MiTy study, the primary composite outcome of neonatal mortality and morbidity (comprising pregnancy loss, preterm birth, birth injury, respiratory distress, neonatal hypoglycemia, and admission to intensive care for > 1 day) was similar with or without metformin added to the insulin [1]. There were no differences in gestational age at birth, shoulder dystocia, or hyperbilirubinemia and also neonatal length of hospital stay was similar between the groups. However, infants exposed to metformin were often smaller and thinner for gestational age, compared with those who were not exposed to metformin: 30 (13%) infants in the metformin group and 15 (7%) in the placebo group were SGA (RR 1.96; 95% CI 1.10–3.64; *p* = 0.026); mean birth weight 3156 ± 742 g versus 3375 ± 742 g; *p* = 0.002). In a recent subsequent analysis of MiTy study, the presence of a comorbidity (chronic hypertension and/or nephropathy) (OR 3.05; 95% CI 1.58–5.81) and metformin use (OR 2.26; 95% CI 1.19–4.74) have been identified as independent predictors of SGA [69].

Moreover, a much higher absolute risk of SGA was observed in women receiving metformin with comorbidity compared with women receiving metformin without comorbidity (25.0% vs. 9.8%). Based on this observation the authors conclude that, with the aim of reducing SGA, it is reasonable to be cautious in the use of metformin in women with T2DM and chronic hypertension or nephropathy in pregnancy.

### Long-term infant outcomes

Recently, the MiTy Kids study evaluated children up to 2 years of age of mothers with type 2 diabetes who participated in the MiTy trial. The primary outcome was the measure of adiposity by the mean BMI Z score and the mean sum of skinfolds thickness (triceps, subscapular, suprailiac) [70]. There was no difference in BMI Z score in the metformin-exposed infants versus the placebo at 24 months of age (0.84 vs. 0.91, mean difference 95% CI 0.07 [− 0.31 to 0.45]; *p* = 0.72), or for skinfolds thickness (23.0 vs. 23.8, mean difference 95% CI 0.8 [− 0.7 to 2.3]; *p* = 0.31). When the BMI trajectories were analyzed, males of metformin group reached a significantly (*p* = 0.048) higher peak than the placebo group at 6 months that continued to be higher at 12 and 18 months, while at 24 months the trajectory was similar. In a small subgroup of children at 24 months the fasting glucose was significantly higher in the metformin group than the placebo group (4.8 mmol/L vs. 4.1 mmol/L; *p* = 0.0090), whereas the other metabolic parameters were similar [70].

In Table 12 are summarized the effects on neonatal and infant outcomes of metformin compared to control group of T2DM women. Table 13 shows main maternal and infant outcomes in T2DM women treated with metformin during pregnancy.

**Table 12:** Neonatal and infant outcomes in T2DM women treated with metformin during pregnancy

	T2DM women
Newborns	
Birth weight	Lower
LGA/Macrosomia	Decreased
SGA	Increased
Hypoglycemia	Decreased
Neonatal intensive care unit admission	No significant difference
Infants	
BMI	No significant difference
Fasting glycemia	Increased

LGA, large for gestational age; SGA, small for gestational age; BMI, body mass index

**Table 13:** Main maternal and infant outcomes in metformin treated women with T2DM

	Maternal outcomes	Infant outcomes
Type 2 diabetes	Reduced maternal weight gain Decreased risk of maternal hypoglycemia Better metabolic control during the later stages of pregnancy	Decreased risk of neonatal hypoglycemia Decreased LGA and reduced body fat of neonates Increased risk for SGA in women with comorbidities as chronic hypertension or nephropathy in pregnancy 2 years old children have similar BMI Z score and skinfolds thickness

## Metformin and breastfeeding

Breastfeeding should be encouraged for all women, and it may be even more important for women with GDM or T2DM given the evidence that breastfeeding may be beneficial in reducing the risk of childhood obesity and insulin resistance. There has been reluctance to use metformin during the breastfeeding period due to some reports of presence of metformin in the breast milk and lack of safety data [71–73]. In three studies of small sample size, metformin was detected in breast milk with a milk-to-plasma ratio of 0.35–0.71. Infant exposure was calculated at approximately 0.28–0.65% of the maternal dose, with very low or undetectable serum levels in the infant. Thus, the amount of metformin in breast milk is likely clinically insignificant, but an open discussion of the uncertainties with the woman is necessary before prescribing it to the lactating patient.

## Recommendations for the use of metformin during pregnancy

Metformin could be considered in very obese women (BMI > 35 kg/m^2^) to reduce gestational weight gain. There is insufficient evidence to support the use of metformin for women in pregnancy with obesity for improving maternal and infant outcomes. Future large RCTs are needed to increase the quality of evidence, to include women categorized as 'overweight' and to look at metformin in combination with other treatment. Moreover, future studies should consider the pre-conception use of metformin and evaluate the optimal dosage in the obese population. In particular, long-term follow-up of the offspring are necessary. Its prescription should be cautious and personalized. Metformin could be considered particularly in obese-PCOS women to reduce the risk of preterm delivery. Metformin could be use in women undergoing ART treatment in combination with clomiphene citrate in women with PCOS who are clomiphene citrate resistant, anovulatory and infertile, not presenting other infertility factors effective to improve fertility outcomes. Metformin use could be a valid therapeutic option in GDM women with obesity. In severe obese women, metformin may reduce the insulin dose and the gestational weight gain. In case of severe obesity, the risk of caloric restriction for the fetus is low and so the risk for long-term metabolic effect could be low in the offspring. Metformin could better assist metabolic control during the later stages of pregnancy in women with T2DM, countering the clinical effects of escalating insulin resistance, without weight gain or hypoglycemia, and may have potential benefits in the neonatal period, except for tendency for lower birth weights and reduced body fat of neonates. Although there are some data reassuring in children up to 2 years old, long-term follow-up studies of offspring exposed to metformin in utero in women with T2DM are still recommended before routine use. Moreover, particular caution should be exercised in the use of metformin in women with type 2 diabetes and chronic hypertension or nephropathy in pregnancy.

## Conclusion

This updated position statement has been designed to provide a consensus approach and practical advice to healthcare professionals for the use of metformin in pregnancy and in the periconceptional period for different health conditions.

In condition affecting the fertility, as PCOS, metformin use in pre-conception or early in pregnancy may reduce preterm delivery. In case of ART, metformin is useful to improve fertility and reduce OHSS risk. In obese women metformin use in pregnancy is associated with a lower GWG, this does not occurred in the overweight ones. In pregnancy complicated by diabetes (GDM or T2DM), metformin improves maternal glycemic control and may reduce insulin dose.

Neonatal and infant outcomes related to metformin exposure in utero are lacking. While metformin does not appear to affect neonatal outcomes in children born to obese or PCOS women or in women undergoing ART, its use in women with GDM or T2DM is associated with lower birth weight. However, an increased tendency towards overweight-obesity has been observed in children born to women with GDM treated with metformin during pregnancy, later in life (Table 14).

**Table 14:** Metformin effects on maternal/fetal outcomes and offspring metabolic profile

	Maternal outcomes					Fetal outcomes		Offspring
Condition	Fertility	GWG	GDM rate	Insulin need	Preterm delivery	Miscarriage	Birth weight	Metabolic Profile
Overweight	No available data	=	=	NA	/	/	=	=
Obesity	No available data	Reduced	=	NA	/	Reduced	=	=
PCOS	Improved	Reduced	=	/	Reduced	=	=	Impaired
ART (in PCOS women)	Improved	No available data	No available data	NA	No available data	/	/	No available data
GDM	No available data	Reduced	NA	Reduced	=	=	Lower	Impaired
T2DM	No available data	Reduced	NA	Reduced	=	=	Lower	=

GWG, Gestational weight gain. NA, Not applicable. Cells with “/“ indicate inconsistent data

Cells with “ = ” indicate similar results to comparator

Despite specific indications are suggested for women with obesity, PCOS, ART, GDM and T2DM based on the extensive specialist-generated literature (Box 1), more research is required specifically on the long-term effects of in utero exposition to metformin.

**Box 1:** Authors indications for metformin use in pregnancy

Condition	Indications
Pregnancy complicated by obesity	Metformin could be considered in very obese women (BMI > 35 kg/m^2^) to reduce weight gain. Its prescription should be cautious and personalized
Pregnancy in PCOS women	Metformin could be considered to reduce preterm delivery particularly in obese women with BMI > 30 kg/m^2^ and in women who undergo ART
Pregnancy obtained by assisted reproduction technology	Metformin could be considered in combination with clomiphene citrate to improve fertility outcomes in women with PCOS who are clomiphene citrate resistant, anovulatory and infertile, not presenting other infertility factors effective
Pregnancy complicated by GDM	Metformin use could be a valid therapeutic option in obese GDM women to reduce GWG. In women with severe obesity metformin may reduce the insulin dose and the GWG
Pregnancy complicated by T2DM	Metformin could be considered in obese women to reduce insulin dose and GWG or in women unable to manage insulin therapy
	In case of metformin therapy before the conception, suspend and switch to insulin therapy, unless in case of concomitant PCOS (discuss and evaluate individual cases with gynecologist and patient)
	Stop Metformin in any evidence of SGA, above all in women with comorbidities as chronic hypertension or nephropathy in pregnancy
Breastfeeding	Evaluate pros and cons with the patient, informing her of the insufficient amount of data in this regard
